# The Medial Septum as a Potential Target for Treating Brain Disorders Associated With Oscillopathies

**DOI:** 10.3389/fncir.2021.701080

**Published:** 2021-07-08

**Authors:** Yuichi Takeuchi, Anett J. Nagy, Lívia Barcsai, Qun Li, Masahiro Ohsawa, Kenji Mizuseki, Antal Berényi

**Affiliations:** ^1^Department of Physiology, Osaka City University Graduate School of Medicine, Osaka, Japan; ^2^MTA-SZTE ‘Momentum’ Oscillatory Neuronal Networks Research Group, Department of Physiology, University of Szeged, Szeged, Hungary; ^3^Department of Neuropharmacology, Graduate School of Pharmaceutical Sciences, Nagoya City University, Nagoya, Japan; ^4^Neurocybernetics Excellence Center, University of Szeged, Szeged, Hungary; ^5^HCEMM-USZ Magnetotherapeutics Research Group, University of Szeged, Szeged, Hungary; ^6^Neuroscience Institute, New York University, New York, NY, United States

**Keywords:** medial septum, oscillation, oscillopathy, deep brain stimulation, epilepsy, Alzheimer’s disease, anxiety/fear, depression

## Abstract

The medial septum (MS), as part of the basal forebrain, supports many physiological functions, from sensorimotor integration to cognition. With often reciprocal connections with a broad set of peers at all major divisions of the brain, the MS orchestrates oscillatory neuronal activities throughout the brain. These oscillations are critical in generating sensory and emotional salience, locomotion, maintaining mood, supporting innate anxiety, and governing learning and memory. Accumulating evidence points out that the physiological oscillations under septal influence are frequently disrupted or altered in pathological conditions. Therefore, the MS may be a potential target for treating neurological and psychiatric disorders with abnormal oscillations (oscillopathies) to restore healthy patterns or erase undesired ones. Recent studies have revealed that the patterned stimulation of the MS alleviates symptoms of epilepsy. We discuss here that stimulus timing is a critical determinant of treatment efficacy on multiple time scales. On-demand stimulation may dramatically reduce side effects by not interfering with normal physiological functions. A precise pattern-matched stimulation through adaptive timing governed by the ongoing oscillations is essential to effectively terminate pathological oscillations. The time-targeted strategy for the MS stimulation may provide an effective way of treating multiple disorders including Alzheimer’s disease, anxiety/fear, schizophrenia, and depression, as well as pain.

We first describe the anatomy of the medial septum (MS) in section “Anatomy of the Medial Septum.” We then provide information on how the MS regulates oscillatory activities in the brain in section “Roles of the MS in Physiological Oscillations.” In section “The Medial Septum as a Target for Deep Brain Stimulation for Epilepsy Control and Beyond,” we discuss the possibility of the MS as a target of deep brain stimulation (DBS) for controlling oscillopathies (epilepsy, Alzheimer’s disease, anxiety/fear, schizophrenia, depression, and pain).

## Anatomy of the Medial Septum

The septal region is conventionally split into four subregions based on anatomical location: the lateral, medial, posterior and ventral groups. Ample evidence stresses the importance of respecting the distinct nature of the septal region’s subregions. Unfortunately, however, many studies that investigate various septal areas refer to them by using the vague term “septum” and fail to precisely define the actual region within the scope of the study. It is particularly important to separate the medial and lateral septal nuclei because these two regions receive and send different modalities through their afferent and efferent fibers, occasionally to the same brain regions, and these modalities have distinct functional roles in information processing.

In this review, we focus on the medial group referred to as the “medial septum (MS),” which consists of the medial septal nucleus and the diagonal band of Broca.

### Neuronal Populations in the Medial Septum

The chemoarchitecture of the MS allows us to distinguish at least three major neuronal populations: cholinergic, GABAergic, and glutamatergic neurons (Dutar et al., 1995).

There are approximately 10,000 cholinergic neurons, containing the enzyme choline acetyltransferase (ChAT), in the rodent MS (Colom, 2006). They are located mainly at the lateral zone of the MS and some of them are surrounded by parvalbumin (PV)-positive neurons. Activation of the cholinergic neurons results in slow excitation of the glutamatergic neurons in the MS. Double staining techniques identified different subpopulations of the cholinergic neurons, which co-release glutamate, nitric oxide, or neuropeptides (e.g., galanin) along with acetylcholine (ACh) (Melander et al., 1985; Forloni et al., 1987; Sotty et al., 2003).

GABAergic neurons in the MS, present at approximately half the number of the MS cholinergic neurons in rodents, express glutamic acid decarboxylase (GAD) (Colom, 2006). They are relatively large and almost exclusively express GAD67; only a few of them express GAD65 (Castañeda et al., 2005). MS GABAergic neurons form non-overlapping subgroups with intracellular calcium-binding protein expression; each expresses either calbindin (CaBP), calretinin (CR), or PV (Freund, 1989; Kiss et al., 1997). The PV-expressing GABAergic neurons are projection neurons located in the midline zone, while the others are local inhibitory neurons (Ang et al., 2017). The main role of the GABAergic neurons is to synchronize the septal network during the its most characteristic oscillation, the theta rhythm (see section “Roles of the MS in Physiological Oscillations”).

The third neuronal population, approximately 16,000 neurons in rodents, is formed by relatively small glutamatergic neurons of diverse morphology. They express vesicle glutamate transporter 1 and 2 (VGluT1 and VGluT2) and are either projection or local neurons. Upon activation, MS glutamatergic neurons evoke strong and fast excitation of intermingled cholinergic and GABAergic neurons (Manseau et al., 2005; Müller and Remy, 2018). In addition to the strong bidirectional interplay between the cholinergic and GABAergic neurons (Leranth and Frotscher, 1989), immunohistochemical and electrophysiological studies confirmed that glutamatergic interneurons are also extensively interconnected in the intraseptal local networks (Manseau et al., 2005; Huh et al., 2010). They act mainly through α-amino-3-hydroxy-5-methyl-4-isoxazolepropionic acid (AMPA) receptors on their peers, and only to a lesser extent through *N*-methyl-D-aspartate (NMDA) receptors (Manseau et al., 2005). This enables the various functional states necessary to generate the characteristic oscillatory patterns of the MS (Robinson et al., 2016).

Note that so far there is no consensus about the exact number and proportion of these neuronal populations of the MS. Particularly the ratio of GABAergic and cholinergic neurons is still in debate. On one hand, studies reported twice as much cholinergic neurons as GABAergic ones (Brashear et al., 1986; Gritti et al., 1993). On the other hand, others provided data about 1:6 ratio of cholinergic to GABAergic neurons (McGeer et al., 1984; Smith and Booze, 1995). This uncertainty may root in the different antibodies and staining techniques applied (Semba, 2000). The total number of neurons of each population differently vary with age as well. The total number of MS neurons decreases about 30% with aging, whereas the number of MS GABAergic neurons remain stable over time (Bender et al., 1996). It is also noteworthy to mention that these neuronal populations are not completely exclusive. For example, glutamate is used as a local transmitter by MS GABAergic and cholinergic neurons (Gritti et al., 2003). It is reported that MS cholinergic neurons use both ACh and GABA as transmitter in the HPC (Takács et al., 2018). The extent of overlap of the three neuronal populations may depend on the examined species. For example, in mice, Takács et al. showed that almost all MS cholinergic neurons express vesicular GABA transporter as well (Takács et al., 2018). On the contrary, in rats and cats, the overlap between cholinergic and GABAergic neurons is relatively low (below 2% in the entire basal forebrain) (Brashear et al., 1986; Takeuchi et al., 2021a).

### Synaptic Connections of the Medial Septum

**FIGURE 1:**
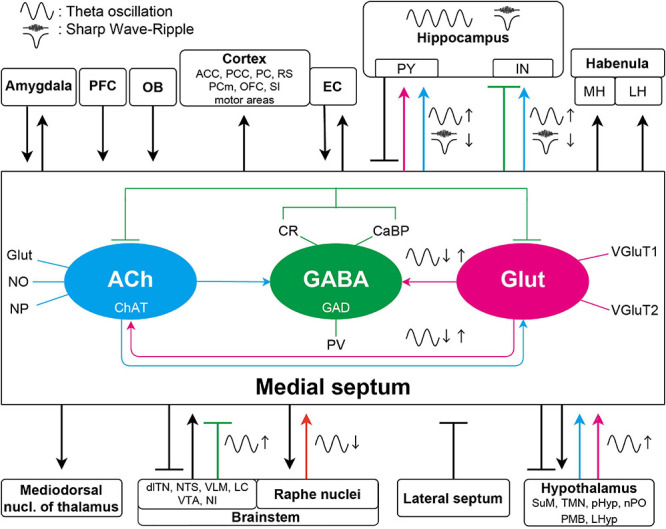
Schematic diagram of the septal connections discussed in this article. The three major neuronal populations maintain a delicate intraseptal network. The medial septal region receives a variety of afferents from the lateral septum, PFC, and forms bidirectional connection with amygdala, EC and a broad range of other neocortical areas, hypothalamus, and brainstem. Unidirectional, overwhelmingly cholinergic efferent fibers are innervating the thalamus and the lateral and medial habenula. For simplicity, specific neurotransmitters and the intraseptal origins or targets of the pathways are only marked in the septohippocampal connections. The medial septum is highly interconnected with the hippocampus as well. These pathways have significant roles in generation or regulation of different hippocampal oscillations. Colored arrows indicate important connections contributing to oscillations in the septo-hippocampal axis. Magenta arrows show glutamatergic, cyan cholinergic, green (blunt) GABAergic, and orange serotonergic innervations. Glutamatergic neurons in the medial septum more likely regulate other theta generating medial septal neuronal populations; however, roles of their projections to the hippocampus in oscillations are largely unknown. The GABAergic neurons have roles in theta generation by disinhibiting the hippocampal pyramidal neurons. The cholinergic connections are not only important in the theta generation, but they also suppress SPW-R generation although whether this suppression acts on the hippocampal pyramidal or interneurons remains elusive. ACC, anterior cingulate cortex; dlTN, dorsolateral tegmental nucleus; EC, entorhinal cortex; IN, interneuron; LC, locus coeruleus; LH, lateral habenula; LHyp, lateral hypothalamus; MH, medial habenula; PCm, medial precentral cortex; NI, nucleus incertus; NTS, nucleus tractus solitarius; OB, olfactory bulb; OFC, orbitofrontal cortex; PCC, posterios cingulate region; PC, piriform cortex; pHyp, posterior hypothalamus; PFC, prefrontal cortex; PMB, posterior mammillary bodies; PY, pyramidal neuron; nPO, nucleus pontis oralis; RS, retrosplenial cortex; SI, substantia innominate; SuM, supramammillary nucleus; TMN, tuberomammillary nucleus; VLM, ventrolateral medulla; VTA, ventral tegmental area.

In the subsequent subsections, we outline the long-range afferent and efferent connections of the MS neurons. In most cases these pathways consist of fibers operating with multiple neurotransmitters, thus we overview them structure by structure rather than focusing primarily on the types of neurotransmitters (Figure 1). Due to its importance, the reciprocal connection of the MS with the hippocampal formation is discussed first. For a discussion of anatomy from a different point-of-view on a transmitter by transmitter basis, see the following articles (Sun et al., 2014; Ang et al., 2017; Müller and Remy, 2018). For example, using combinations of cell type specific Cre-driver mouse lines and monosynaptic rabies viral vectors, Sun et al. showed that 66% of septohippocampal neurons that innervate HPC CaMKIIα-positive cells were cholinergic and 27% of them were GABAergic. They also showed that 67% of septohippocampal neurons that innervate HPC GABAergic neurons were GABAergic, and 12 and 27% of them were cholinergic and glutamatergic, respectively. Note that despite these sophisticated experiments, there still may be overlaps between immunohistochemically identified MS neuronal types.

#### The Septo–Hippocampal–Septal Loop (Septo–Entorhinal–Septal Loop)

The anatomical and functional interplay between the MS and the hippocampal formation is very important in many cognitive functions, including learning and memory. The MS and the hippocampus (HPC) have reciprocal connections establishing a prominent contribution of the MS to HPC theta oscillations. The MS cholinergic, GABAergic, and glutamatergic neurons all project to the HPC cornu ammonis 1 region (CA1).

Projecting axons of all the MS neurons enter the HPC via the fimbria/fornix. The cholinergic projections are nearly 65% of the total MS to HPC projections although the percentage greatly varies depending on the targeted subregion and neuronal type as mentioned above: larger on excitatory neurons than that on inhibitory neurons in CA1 region of the HPC (Frotscher and Léránth, 1985; Klausberger and Somogyi, 2008; Teles-Grilo Ruivo and Mellor, 2013; Sun et al., 2014). A lesion study revealed that MS cholinergic projections reach the dorsal HPC and the medial entorhinal cortex (MEC) via the fornix, whereas they reach the ventral HPC and the lateral entorhinal cortex (LEC) via the fimbria (Mitchell et al., 1982). MS cholinergic neurons for example innervate the HPC CA1 and activate the oriens-lacunosum-moleculare (O-LM) neurons, a subgroup of HPC somatostatin (SST)-positive GABAergic interneurons. The O-LM neurons in turn inhibit the distal dendrites of HPC pyramidal neurons, which inhibits the temporoammonic inputs from the entorhinal cortex (EC) (Reece and Schwartzkroin, 1991; Leão et al., 2012; Haam et al., 2018). On the other hand, other SST-positive GABAergic interneurons in the HPC are capable of controlling the Schaffer collaterals (Müller and Remy, 2018). Therefore, the MS cholinergic innervation can balance inputs of the HPC CA1 in a pathway-specific manner.

A subpopulation of MS GABAergic neurons that expresses PV and hyperpolarization-activated cyclic nucleotide-gated (HCN) channels presumably provides the theta rhythmic drive to the HPC (Varga et al., 2008). The septal PV/HCN GABAergic neurons inhibit and then disinhibit PV-positive GABAergic neurons in the HPC in a theta rhythmic manner (Varga et al., 2008). The activated PV-positive GABAergic neurons subsequently control the activation of principal neurons in the dentate gyrus (DG), the HPC cornu ammonis 3 region (CA3) and the HPC CA1 (Freund and Antal, 1988).

Some MS neurons that co-express neuropeptides project to the HPC. For example, galanin-expressing MS cholinergic neurons project to the ventral HPC (Melander et al., 1985). *N*-Acetylaspartylglutamate-expressing MS neurons project to the dorsal HPC (Forloni et al., 1987).

GABAergic neurons in the stratum oriens of the HPC CA1 form their synapses with dendrites and cell bodies of the MS neurons, which forms a feedback loop (Toth et al., 1993). SST-positive GABAergic neurons of the DG also project to the MS, where they strongly inhibit MS glutamatergic neurons and weakly inhibit MS GABAergic and cholinergic neurons. These hippocampo-septal neurons are strongly activated during sharp wave–ripples (SPW–Rs) (Jinno et al., 2007; Yuan et al., 2017).

Septal glutamatergic efferents reach the HPC CA3, CA1, and the DG (Colom et al., 2005). The septal glutamatergic fibers terminate on the CA1 oriens/alveus interneurons. Experiments on mice have found that the activation of the CA1 oriens/alveus interneurons by the septal glutamatergic fibers accords with the actual running speed of the mice (Freund and Buzsáki, 1996). Therefore, the firing rate and the number of activated MS glutamatergic neurons can predict the future running speed in mice (Fuhrmann et al., 2015). The MS glutamatergic neurons make excitatory synapses on the MS GABAergic neurons and the MS GABAergic neurons then make inhibitory synapses on the GABAergic interneurons in the HPC. Therefore, the activation of MS glutamatergic neurons then increases the firing rate of the CA1 pyramidal neurons by disinhibition through a chain of feedforward inhibition at higher running speed.

The MS also has reciprocal connections with the EC. Septal projections arise from the ventrolateral MS, mostly from the vertical and horizontal limbs of the diagonal band of Broca (VDB and HDB, respectively) (Woolf et al., 1984). The septal efferents run to the EC of both hemispheres and were proved to be mainly cholinergic (Alonso and Köhler, 1984). The septal efferents terminate in layers I and II in the MEC and LEC. A part of the EC neurons show monosynaptic and/or polysynaptic GABA_A_ receptor-mediated responses upon optogenetics activation of axon terminals of MS cholinergic neurons, which suggests that the MS cholinergic neurons are able to co-transmit GABA with ACh (Desikan et al., 2018; Takács et al., 2018). The MS cholinergic neurons target mainly the layer I and II 5-HT_3_ receptor-positive interneurons in the MEC, but other layer I and II LEC neurons were also regulated by the MS through the 5-HT_3_ receptor-positive interneurons in these layers (Desikan et al., 2018).

#### Afferent Innervation of the Medial Septum

The MS receives many neuromodulatory afferents including cholinergic ones from the dorsolateral tegmental nucleus, adrenergic ones from the locus coeruleus, serotonergic ones from the raphe nuclei, dopaminergic ones from the ventral tegmental area (VTA), histaminergic ones from the hypothalamus, and GABAergic ones from the HPC and the lateral septum (LS) (Segal, 1982; Semba et al., 1988). The ascending fibers from the brainstem to the MS mainly pass through the medial forebrain bundle. They not only innervate the MS neurons with neuromodulatory inputs, but also pass through the MS to target the LS and the HPC (Risold and Swanson, 1997a, b).

The noradrenergic fibers, originating from the ventrolateral medulla (A1 cell group) and nucleus tractus solitarii (A2 cell group) of the brainstem, project to the MS and modulate septal gonadotropin hormone-releasing hormone (GnRH)-secreting neurons (Kaba et al., 1983; Kim et al., 1987; Wright and Jennes, 1993; Hosny and Jennes, 1998). The locus coeruleus noradrenergic system also reaches the MS (Lindvall and Stenevi, 1978).

The raphe complex is one of the midbrain nuclei. It sends serotonergic fibers to the MS and the LS (Fuxe, 1965; Conrad et al., 1974). These afferents originating from the median raphe nucleus (MRN) desynchronize hippocampal electroencephalography (EEG) (Assaf and Miller, 1978). This is an indirect effect via the MS through the excitation of the GABAergic cells in the MS expressing 5-HT_2A_ receptors, rather than a direct serotonergic influence of the HPC (Leranth and Vertes, 1999).

Combined retrograde studies proved that the A10 dopaminergic neurons of the VTA send ascending projections to the diagonal band of Broca and the LS (Swanson, 1982; Kalivas, 1985).

The LS is one of the key input areas of the MS (Swanson and Cowan, 1979). The dorsal part of the LS projects almost exclusively to the nucleus of the diagonal band, whereas the intermediate and ventral parts of the LS project to the whole extent of the MS. Leranth et al. (1992) highlighted that the LS projections targeting the MS are sparse. The LS rather projects denser on the hypothalamus and the hypothalamus projects back to the MS (Leranth et al., 1992).

The supramammillary nucleus (SuM) of the hypothalamus was identified as a modulator/driver of the HPC theta rhythm generation during some behavioral tasks and during urethane anesthesia as well (Kirk and McNaughton, 1991; Kirk and McNaughton, 1993; Kirk et al., 1996). Neurons in the SuM fire in a theta burst manner in response to non-rhythmic inputs from the reticular formation (Kocsis and Vertes, 1994). The CR-positive aspartate/glutamatergic neurons of the SuM then excite the MS cholinergic neurons and the HPC pyramidal neurons (Frotscher and Léránth, 1985, 1986; Leranth and Kiss, 1996; Kiss et al., 2000). These indicate a complex supramammillary–septal–hippocampal loop: the recipient HPC principal neurons of the MS terminate on the CaBP-positive GABAergic neurons of the LS, which close the circuit by providing feedback to the SuM CR neurons (Risold and Swanson, 1996). Moreover, the GABAergic MS neurons and the SuM CR neurons directly innervate the LS (Leranth et al., 1992; Leranth and Kiss, 1996).

The histaminergic neurons of the hypothalamus found in the tuberomammillary nucleus (TMN) innervate the GABAergic and MS cholinergic neurons with particularly dense axon terminals (Panula et al., 1989). Their activity shows clear circadian rhythmicity (Mochizuki et al., 1992). It was shown to maintain wakefulness, because lesion of the TMN histaminergic neurons resulted in increased slow-wave sleep and hypersomnolence (Lin et al., 1989). The histaminergic innervation to the MS has roles in learning and memory (Xu et al., 2004b).

Other hypothalamic regions, such as the posterior hypothalamus and the nucleus pontis oralis also send afferents to the MS. These afferents are cholinergic and act primarily on the muscarinic receptors of the MS. Their electrical stimulation can evoke theta oscillations through the activation of the MS (Bland et al., 1994). Diencephalic afferents were also identified from the lateral preoptic and lateral hypothalamic areas. The premammillary and supraoptic nuclei project to the caudal and rostral parts of the MS, respectively (Swanson, 1976; Saper et al., 1979).

A reciprocal connection between the MS and the nucleus incertus (NI) was proved by retrograde labeling of NI. These projections are passing through the MS (Goto et al., 2001; Olucha-Bordonau et al., 2003) and may modulate the HPC theta rhythms by a potential mediator peptide, relaxin-3 (Ma et al., 2009b), which is co-released with GABA (Tanaka et al., 2005). An inhibitory feedback projection was also described from the MS to the NI, which modulates the ascending afferents of NI (Sánchez-Pérez et al., 2015).

Tract-tracing experiments identified further afferents of the MS from the amygdala and the prefrontal cortex (Russchen et al., 1985; Sesack et al., 1989; Hurley et al., 1991).

#### Efferent Projections From the Medial Septum

The efferent connections of the lateral and medial parts of the MS are topographically organized. Regarding the hippocampal formation, the lateral part of the MS preferentially projects to the ventral parts of the subiculum, the HPC, the MEC and the LEC. On the other hand, the medial parts of the MS mainly project to the dorsal and ventral HPC, and the dorsolateral EC (Gaykema et al., 1990). The lateral and intermediate parts provide efferents to the olfactory regions, taenia tecta, medial and cortical amygdaloid nuclei, and the LEC (dorsolateral and ventrolateral ECs). The medial part of the MS sends fibers to the vertical diagonal band; anterior cingulate cortex; retrosplenial cortex; medial precentral and motor areas; indusium griseum; olfactory regions; and the orbital prefrontal cortex (Woolf et al., 1984; Woolf and Butcher, 1986).

Investigation of the cholinergic system and projections from the pontomesencephalic tegmentum to the thalamus and basal ganglia revealed information about the septal efferent connections (Woolf et al., 1984). Woolf et al. found that the olfactory bulb receives almost all MS fibers from the HDB (Woolf et al., 1984). Later it was identified that most of the cholinergic septal efferents originate from the medial half of HDB, while most of the non-cholinergic efferents arise from the lateral half of the HDB. Approximately 30% of the HDB projection neurons are GAD-positive (Záborszky et al., 1986). Purely cholinergic projections were described from the caudodorsal medial septal nucleus and both limbs of the diagonal band to the amygdala, from the HDB to piriform cortex, and from the ipsilateral MS to the magnocellular preoptic/ventral pallidal area (Woolf et al., 1984; Woolf and Butcher, 1986). Cholinergic projections of VDB origin also innervate the substantia innominata (Parent et al., 1988). Cholinergic efferents from MS innervate the posterior cingulate region (Woolf et al., 1984), as well as the rostral anterior cingulate cortex; this latter pathway seems to be involved in maintaining anxiety during chronic pain, independently from the septo-hippocampal pathway (Jiang et al., 2018a, b). These fibers form synapses with GABAergic interneurons in the cingulate and retrosplenial cortices (Semba, 2000).

Although research interest regarding the efferent connections of the MS was biased toward the cholinergic system and its role in attention, the MS GABAergic and glutamatergic projections should not be neglected. A significant portion of the non-cholinergic fibers project to the thalamus, the hypothalamus and the brainstem. The cholinergic fibers targeting cortical areas are frequently coupled with GABAergic fibers, but cholinergic axons outnumber the GABAergic fibers in these bundles (Semba, 2000).

The medial habenula receives GABAergic and glutamatergic inputs from the MS (Choi et al., 2016), in addition to the cholinergic innervation described by Woolf and Butcher (Woolf and Butcher, 1986). Results of the former study indicated that septal GABAergic input alone was able to modulate the firing of medial habenula neurons via activation of GABA_A_ receptors, combined with a delayed inhibition through GABA_B_ receptors. These septal fibers are under massive control in the medial habenula by endocannabinoid signaling, which is hypothesized to be important in anxiety and depression (Vickstrom et al., 2020). The glutamatergic septal inputs to the lateral habenula and to the preoptic area have key roles in inducing place aversion and enhanced locomotion, respectively (Zhang et al., 2018).

Horseradish peroxidase injection in the posterior mammillary bodies indicated a direct connection with the MS. Anterograde tract tracing of the lateral and vertical diagonal band resulted in labeled fibers which were passing through the medial forebrain bundle and innervating the SuM before they enter the mammillary bodies (Meibach and Siegel, 1977).

The MS sends mostly non-cholinergic efferent projections to the raphe nuclei. The MS fibers reach the basal mesencephalon and the rostro-medial pontine nuclei before they project to the caudal part of the dorsal raphe and the central superior raphe nucleus. The VDB fibers reach the raphe nuclei by two routes: some of them enter both raphe nuclei by passing through the basal mesencephalon whereas the others reach the dorsal raphe through the pedunculopontine nucleus (Kalén and Wiklund, 1989). Importantly, DBS of the MS in humans was found effective to relieve chronic pain (see section “Roles of the MS in Physiological Oscillations”). The exact pathway responsible for this analgesic remains unclear; however, the descending inhibitory pathway from the MS to the dorsal horn neurons of the spinal cord via the raphe nucleus may play a key role (Hagains et al., 2011).

It is worth mentioning some other target brain areas of the MS neurons. The MS cholinergic and GABAergic neurons project to the mediodorsal nucleus of the thalamus. They might have significant roles in modulating thalamic excitability (Gritti et al., 1998). The MS GABAergic neurons project to the lateral hypothalamus as well. This pathway presumably regulates food intake (Sweeney and Yang, 2016).

## Roles of the Ms in Physiological Oscillations

There are three prominent physiological oscillations in the septo-hippocampal axis: theta, gamma, and SPW–Rs (Colgin, 2016). The MS has been indicated to have an important role in governing these physiological oscillations with massive interconnection with the hippocampal formation (Dutar et al., 1995; Müller and Remy, 2018), although the exact origin of these oscillation is still in debate.

### Generation and Modulation of Theta Oscillations

#### Contributions to Theta Oscillations

Theta oscillations are 4–12 Hz rhythms with a relatively high amplitude dominating the HPC local field potential (LFP). The MS is considered to be a key structure in generating theta oscillations (Petsche et al., 1962). They emerge during active exploration, voluntary movements (e.g., walking, running, jumping), rapid eye movement (REM) sleep and certain brain states related to arousal (e.g., freezing behavior in an anxious environment) (Vanderwolf, 1969; Bland, 1986). Type 1 theta (fast) and type 2 theta (slow) are distinguished based on their sensitivity to atropine (a muscarinic ACh receptor antagonist) (Sainsbury and Montoya, 1984): Type 1 and 2 theta oscillations are atropine-resistant and atropine-sensitive, respectively. Type 1 theta is associated with spatial navigation and movement, whereas type 2 theta is associated with arousal and anxiety on sensory salience (Sainsbury and Montoya, 1984; Buzsáki, 2002). *In vitro* and *in silico* experiments suggest that theta oscillations can be intrinsically generated in the HPC inhibitory and excitatory networks (Buzsáki, 2002; Goutagny et al., 2009; Neymotin et al., 2011). However, extensive in vivo studies have suggested that external drivers, including those from the MS, are involved in the theta oscillations as well (Wang, 2002). For example, lesions of the MS abolish theta oscillations in the septo-hippocampal axis (Partlo and Sainsbury, 1996) and cooling of the MS slows the theta rhythms (Petersen and Buzsáki, 2020).

Both MS GABAergic and cholinergic neurons contribute to the theta rhythms (Smythe et al., 1992; Yoder and Pang, 2005; Ma et al., 2012). MS GABAergic neurons target HPC interneurons exclusively (Unal et al., 2015). Therefore, their burst firing disinhibits HPC pyramidal neurons in a theta phase-locked manner (King et al., 1998; Borhegyi et al., 2004). A subpopulation of these HPC-targeting MS GABAergic neurons, which express PV and HCN channels, specifically drives theta rhythm in the HPC (Varga et al., 2008; Hangya et al., 2009). In vitro studies have suggested that hyperpolarization-activated (H) currents can be identified as pacemaker currents in the MS GABAergic neurons. The H currents presumably contribute rhythmic activity of the PV/HCN MS GABAergic neurons along with network-level interactions and then theta oscillations in the septo-hippocampal axis. This suggestion arises because *in vivo* injection of a H current blocker into the MS did indeed reduce discharge frequency of the PV/HCN MC GABAergic neurons and power of theta oscillations in the HPC (Xu et al., 2004a; Varga et al., 2008). The intervention to MS GABAergic neurons affects theta oscillations in the MEC as well, there MS GABAergic neurons project (see section “Theta Oscillations and Cognitive Maps”). The synaptic transmission at the synapses formed between these MS GABAergic neurons and HPC GABAergic interneurons exhibits a rapid recovery of short-term depression by excitation trains, which enables highly efficient transmission at the synapses even with frequent transmissions (Yi et al., 2021). The HPC to MS feedback projections via the HPC GABAergic neurons also contribute to the theta oscillations in the septo-hippocampal axis (Kang et al., 2017).

MS cholinergic neurons target both HPC pyramidal and GABAergic interneurons (Sun et al., 2014). They fire in a more irregular way compared with MS GABAergic neurons, but their firings are still phase-locked to theta oscillations (King et al., 1998). Selective destruction of the MS cholinergic neurons leads to a decrease of the theta amplitude in the dorsal HPC, leaving the frequency of the oscillation intact (Zheng and Khanna, 2001). Optogenetic activation of the MS cholinergic neurons increases the theta power in mice (Vandecasteele et al., 2014).

As noted in section “Anatomy of the Medial Septum,” the MS receives synaptic inputs from brain regions outside the septo-hippocampal axis, and the inputs to the MS regulate theta oscillations in the septo-hippocampal axis as well. For example, serotonergic projections from the MRN alter the firing pattern of the MS neurons, which results in the desynchronization of theta oscillations in the HPC (Leranth and Vertes, 1999). Serotonin depletion in the MS by 5,7-dihydroxytryptamine increases theta frequency, which facilitates spatial learning (Gutiérrez-Guzmán et al., 2017). Electrical or optogenetic activation of the NI also provokes theta oscillations in the HPC via MS GABAergic neurons (Albert-Gascó et al., 2018; Lu et al., 2020).

It is important to note that the above observations were almost entirely made with rodents. Therefore, translation of the findings to clinical studies needs careful consideration. The septo-hippocampal connections in primates are very similar to those of rodents (Gulyás et al., 1991). However, to date there are very few human studies about the exact anatomy of the MS and its projections. Due to the obvious ethical considerations, mainly epilepsy patients are involved in the studies, where the network-level functions might have already been altered. Previously, only one type of theta in the human HPC was known, with a lower frequency than those in rodents (Jacobs, 2014). Recently Goyal et al. (2020) identified distinct faster (∼8 Hz) and slower (∼3 Hz) theta oscillations. The faster oscillations are more evident in the posterior HPC (equivalent to the dorsal HPC of rodents) and their power is proportional to movement speed. The slower oscillations are more prevalent in the anterior HPC (equivalent to the ventral HPC in rodents) without any relationship to movement speed. Furthermore, another study proved that theta–gamma phase amplitude coupling (PAC) also exists in humans, and this supports memory (Vivekananda et al., 2021). These studies indicate that the physiological roles of theta oscillations are similar in rodents and humans.

#### Theta Oscillations and Learning and Memory

The HPC is involved in cognitive functions, including learning and memory (O’Keefe, 1993; Bird and Burgess, 2008; Aronov et al., 2017; Korotkova et al., 2018; Mastrogiuseppe et al., 2019). Theta oscillations in the septo-hippocampal axis are thought to support learning and memory because disruption of the theta oscillations by MS inactivation impairs HPC-dependent memory as well (Mizumori et al., 1990; Bannerman et al., 2004; Lecourtier et al., 2011; Wang et al., 2015).

Disruptions of either MS GABAergic or cholinergic neurons, which impair theta oscillations in the septo-hippocampal axis, impair HPC-dependent memory as well. For example, intraseptal muscimol injection impaired memory in a spontaneous alternation and continuous multiple trial inhibitory avoidance task; the memory impairment was blocked by intra HPC injection of bicuculline (Krebs-Kraft et al., 2007). This suggests that septohippocampal GABAergic neurons support the memory. In addition, chemogenetic silencing of MS GABAergic terminals in the HPC disturbed memory retrieval (Sans-Dublanc et al., 2020). Furthermore, optogenetic silencing of these neurons specifically in REM sleep prevented memory consolidation (Boyce et al., 2016). Selective pharmacological lesion of MS GABAergic neurons impaired extinction of learned avoidance in rats (Pang et al., 2011).

The MS cholinergic neurons along with theta oscillations are known to be essential for memory because selective lesion of the cholinergic neurons by 192 IgG-saporin resulted in spatial memory impairments (Easton et al., 2011; Jeong et al., 2014). Sugisaki et al. (2011) showed that the MS cholinergic neurons are crucial for spike timing dependent plasticity in the HPC CA1.

The theta oscillations in the septo-hippocampal axis are important for development of the memory circuits during postnatal periods (Reh et al., 2020). Random optogenetic activation of the MS during postnatal days 21–25 to disrupt HPC theta oscillations caused spatial learning deficits later (in postnatal days 50–60) in rats (Kloc et al., 2020).

#### Theta Oscillations and Cognitive Maps

The MS-governed theta oscillations in the septo-hippocampal axis precisely organize firings of HPC and MEC neurons by providing a temporal window, in which the neurons fire in a phase-locked manner (O’Keefe and Recce, 1993; Tsanov, 2017). The temporally organized firings of HPC and MEC neurons implement cognitive maps including spatial representation by place and grid cells, which thereby enables spatial navigation by path integration with head-direction and speed cells (O’Keefe, 1976; Hafting et al., 2005; McNaughton et al., 2006; Iwase et al., 2020). The time window of the theta oscillations also enables HPC and MEC neurons to implement time-compressed representations of the cognitive maps by phase precession (O’Keefe and Recce, 1993; Buzsáki and Llinás, 2017). Pharmacological inactivation of the MS diminished the theta oscillations and the precisely organized firing patterns of the HPC and MEC neurons (e.g., disruption of spatially periodic firing of the grid cells) (Koenig et al., 2011; Wang et al., 2015), which in turn caused distortion of cognitive maps implemented in the septo-hippocampal axis. The distortion was on the spatial (physical) cognitive map in the brain, which might be analogous to distortion of mental cognitive maps in patients with psychiatric disorders (e.g., schizophrenia). Along with the theta oscillations, the MS provides speed (movement velocity) information to the HPC and the MEC, which is essential for path integration within the spatial cognitive map (which might be used in other cognitive maps) (Hinman et al., 2016; Justus et al., 2017). The glutamatergic and GABAergic neurons in the MS convey the speed information to the HPC and the MEC with theta oscillations (Kaifosh et al., 2013; Bender et al., 2015; Fuhrmann et al., 2015) and inactivation of the MS disrupted the representations of speed signals there, resulting in poor performance of spatial tasks (Hinman et al., 2016; Jacob et al., 2017) (see section “Contributions to Theta Oscillations” as well). Thus, the normal septal activity providing theta oscillations to the HPC–EC loop is presumably crucial for recognizing navigation (where we are now) in the cognitive maps implemented by neuronal firings in the brain.

#### Theta Oscillations and Anxiety/Fear

The type 2 theta oscillation arises in the septo-hippocampal axis in anxious environments or with novelty (Sainsbury and Montoya, 1984). The anxiety signal is related to the ventral HPC, and is represented as synchrony with the medial prefrontal cortex and the amygdala (Kjelstrup et al., 2002; Bannerman et al., 2003; McEown and Treit, 2009; Adhikari et al., 2010; Likhtik et al., 2014). Lesion or inactivation of the MS disrupts the type 2 theta oscillation and decreases anxiety behaviors in rats (Menard and Treit, 1996; Bannerman et al., 2004; Degroot and Treit, 2004). The anxious environment-induced type 2 theta oscillation and associated anxiety were shown to be dependent on the MS cholinergic neurons because lesion or inactivation of MS cholinergic neurons reduced them (Nag et al., 2009). They are also regulated by phospholipase C β4 in the MS and a T-type voltage-gated calcium channel (Cav 3.2), which is highly expressed in the septo-hippocampal axis (Shin et al., 2009; Gangarossa et al., 2014; Arshaad et al., 2021). The anxiety-related theta oscillations in the septo-hippocampal axis are externally regulated. For example, activation of the MRN diminished the theta oscillations and was anxiolytic (Hsiao et al., 2013). In contrast, inhibition of the MRN via activation of local GABAergic interneurons in the nucleus enhanced the theta oscillations and promoted anxiogenic outcomes (Hsiao et al., 2012).

### Modulation of Gamma Oscillations

Gamma oscillations are 25–150 Hz low-amplitude rhythms in the LFP (Bragin et al., 1995; Buzsáki and Wang, 2012; Colgin, 2016). Gamma oscillations in the hippocampal formation are classified into several frequency bands (e.g., slow, mid, fast gamma) (Colgin et al., 2009; Schomburg et al., 2014; Lasztóczi and Klausberger, 2016). The distinct gamma oscillations give rise to different mechanisms in a pathway-specific manner and coordinate neuronal ensembles in the upstream and downstream brain regions (Schomburg et al., 2014; Fernández-Ruiz et al., 2017). They are involved in different information processing (e.g., velocity, where, what) (Zheng et al., 2015; Fernández-Ruiz et al., 2021). The MS-governed theta oscillations provide temporal windows for temporal organization of these frequency-, pathway-, and function-specific gamma oscillations in theta cycles in a phase–phase coupling or phase–amplitude coupling manner (Canolty and Knight, 2010; Belluscio et al., 2012; Schomburg et al., 2014). The MS is essential for the cross-frequency coupling (Neymotin et al., 2011; Radiske et al., 2020), which is thought to be important in learning and memory (Lisman and Buzsáki, 2008; Tort et al., 2009; Amemiya and Redish, 2018).

### Modulation of Sharp Wave–Ripples

Sharp wave–ripples (SPW–Rs) are episodes caused by highly synchronous excitation in the HPC, each of which consists of a single high-amplitude wave followed by a fast 110–250 Hz oscillatory event at the pyramidal cell layer (Buzsáki, 2015). They occur during awake immobility, consummatory behaviors and slow-wave sleep, and are associated with memory consolidation and replays (Girardeau and Zugaro, 2011; Buzsáki and Silva, 2012; Pfeiffer and Foster, 2013; Buzsáki, 2015).

It is known that the majority of the MS neurons are inhibited during SPW–Rs, when the HPC neurons fire in a high probability (Dragoi et al., 1999). On the other hand, when MS-governed theta oscillations dominate in the septo-hippocampal axis, SPW–Rs do not occur (Buzsáki and Silva, 2012). The switch of the two exclusive states is controlled by the MS cholinergic inputs to the HPC because optogenetic activation of the MS cholinergic neurons enhanced theta oscillations and suppressed occurrence of SPW–Rs in the HPC (Vandecasteele et al., 2014). The additional theta enhancement and ripple suppression by optogenetic activation of MS cholinergic neurons were evident in anesthetized (sleeping) mice. The additional modulations by the cholinergic signaling can be observed during awake quiescent states as well but not during awake moving states, when awake ripples do and don’t occur, respectively (Vandecasteele et al., 2014). In the quiescent states, endogenous muscarinic ACh receptors do not seem saturated because systemic administration of pilocarpine, a muscarinic agonist, or donepezil, an AChE inhibitor, still abolishes occurrence of ripples in head-fixed awake mice (Norimoto et al., 2012). In contrast, muscarinic ACh receptors are presumably saturated in the HPC during the moving states. Vandecasteele et al. suggested that SPW–Rs are initiated by the excitatory recurrent collaterals of CA3 pyramidal neurons, when the subcortical controlling neurotransmitters, including ACh, are reduced (Vandecasteele et al., 2014). ACh presumably restricts this SPW–R initiation and its spread by inhibiting the glutamate release on the presynaptic terminal of CA3 neurons. The MS is not required for generation of SPW–Rs.

## The Medial Septum as a Target for Deep Brain Stimulation for Epilepsy Control and Beyond

As we described in the previous sections, the MS governs physiological oscillatory brain activities, which are closely related to normal functions of the brain. In particular neurological and psychiatric disorders where normal oscillations are disrupted, the normal functions of the brain are also disrupted (Mathalon and Sohal, 2015; Braun et al., 2018; Takeuchi and Berényi, 2020). If the disrupted oscillations are governed by the MS, patterned stimulation of the MS with DBS technology (Kringelbach et al., 2007; Krauss et al., 2020) may be able to compensate for the disrupted septal-governed oscillations or mitigate abnormal oscillations, and might be able to modulate symptoms of those oscillopathies as well (Takeuchi and Berényi, 2020). In addition, recent results of clinical trials of gamma frequency entrainment of the brain by sensory stimulation in dementia patients indicate that oscillations can be a therapeutic target (Chan et al., 2021). Stimulation of the MS affects oscillations in many brain regions and then various functions via its widespread efferents (“proxy stimulation”; Takeuchi et al., 2021a) (and possibly via afferents as well). In addition, the stimulation of the MS is effective to modulate oscillations in the limbic system. For example, studies showed that electrical and optogenetic stimulation of the MS is robustly transmitted to the HPC at the same frequency that is applied within the delta to gamma frequency bands (Sinel’nikova et al., 2009; Zutshi et al., 2018; Takeuchi et al., 2021a). Earlier study of the MS stimulation in humans in 1950 reported a high complication rate, but it was likely related to the inexperience of the teams with depth electrode placement (Baumeister, 2000; Fisher, 2015). A more modern study on human MS stimulation reported good tolerance of the MS stimulation, with no side effects reported (Schvarcz, 1993). In general, the identified complication rate of modern DBS treatments for Parkinson’s disease is 6.5% for any complications (McGovern et al., 2013). It should be noted that implantation of depth electrodes in humans must be carefully judged with an acceptable risk–benefit ratio.

In this section, we briefly review pathological changes of the MS in epilepsy and other oscillopathies, and possible MS-mediated intervention strategies for these oscillopathies. Note that the roles of MS in diseases discussed here are mainly based on results of experiments using animal models. Their validity for human disorders is uncertain and thus the proposed therapeutic strategies are hypothetical.

### Epilepsy

Epilepsy is a neurological disorder characterized by an enduring predisposition to generate epileptic seizures (Fisher et al., 2014). Epileptic seizures come with hypersynchronous neuronal activities (seizure waves) and loss of consciousness and/or convulsion. Approximately 1% of the world’s population have epilepsy and one-third of people with epilepsy are refractory for pharmaceutical treatments (Kwan et al., 2011; Chen et al., 2018). Temporal lobe epilepsy (TLE) is one of the most refractory types of epilepsy. In TLE, the HPC is typically a focus of seizures. Uncontrolled seizures of TLE may become secondarily generalized, which increases risks of sudden unexpected death in epilepsy (Bone et al., 2012; Massey et al., 2014). DBS has been investigated for controlling seizures of drug-resistant epilepsy (Li and Cook, 2018). Stimulation of the anterior nucleus of the thalamus, the centromedian nucleus of the thalamus, and the HPC, have been found to be effective in reducing seizures in drug-resistant epilepsy patients. A few clinical studies have been conducted to study anti-epileptic effects of stimulation of the cerebellum and the nucleus accumbens (NAc). Fisher has predicted possible benefits of MS stimulation for drug-resistant epilepsy based on evidence from septum stimulation in animal models of epilepsy and clinical studies on septum stimulation in schizophrenia and pain patients (Fisher, 2015). However, there have been no clinical studies of MS stimulation for epilepsy patients to date. Here, we summarize the recent evidence that MS stimulation can alleviate symptoms of epilepsy in animal studies and propose a closed-loop MS stimulation strategy for more sophisticated therapy.

In the healthy septo-hippocampal axis, the rhythms in the MS (LFP and unit firings) are very coherent and strongly coupled to the HPC, mainly in the theta frequency range (5–12 Hz). This coherent coupling is disrupted in epileptic conditions of animals. The amplitude of the theta oscillation in the septo-hippocampal axis is significantly reduced in animal models of TLE (Colom et al., 2006; Kitchigina et al., 2013). This disruption in theta oscillation is due to both changes in functional coupling between the MS and the HPC and anatomical alterations in the septo-hippocampal axis (e.g., coherence, theta, unit-theta and unit-epileptic spike phase-locking are altered; and there is loss of SST-positive interneurons in the DG) (Colom et al., 2006; García-Hernández et al., 2010; Hofmann et al., 2016). The reduction of the neuronal connections between the MS and HPC was also found in TLE patients (Wang et al., 2020b). The hypothesis is that the MS reduces the seizure susceptibility of the HPC by generating the theta rhythm in the septo-hippocampal axis (Fisher, 2015). In fact, theta activity in the MS (either spontaneous or sensory-evoked) has been shown to abolish epileptiform events in the HPC of animals (Kitchigina and Butuzova, 2009).

**FIGURE 2:**
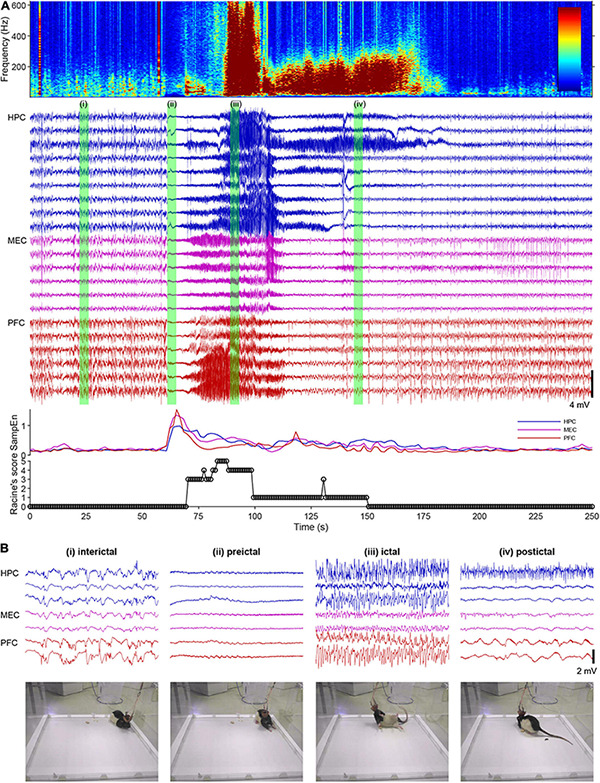
A spontaneous seizure (ictal) episode with convulsion of a rat kainate-induced chronic model of temporal lobe epilepsy (TLE). **(A)** Local field potentials (LFPs) in the hippocampus (HPC), the medial entorhinal cortex (MEC), and the prefrontal cortex (PFC), time-frequency spectrogram of the third HPC channel, sample entropy, and behavioral manifestations over an ictal episode. Racine’s score: 1, mouth and facial movements; 2, head nodding; 3, forelimb clonus; 4, rearing; 5, rearing and falling (Racine, 1972). **(B)** Enlarged LFPs and snapshots of video monitoring green-labeled in **(A)**.

Epileptic brains have at least two distinct stable oscillatory states: interictal (resting) and ictal (hypersynchronous) states (Takeuchi and Berényi, 2020). These states have been validated by in vivo animal and human recordings and in silico modeling studies (Jirsa et al., 2014; Kalitzin et al., 2019). Practically, four brain states can be determined across a spontaneous seizure episode: interictal, preictal, ictal, and postictal suppression periods (Figure 2). For intelligent intervention of epilepsy via MS stimulation, stimulus parameters (intensity, pulse width, frequency, inter-burst interval etc.) and how the stimulus is delivered (e.g., open-loop or closed-loop) should be determined or switched dependent on the targeted states. This is because the open-loop MS stimulation at a certain frequency (e.g., theta) decreases seizure susceptibility during the interictal state whereas it induces pro-seizure effects during the ictal state in rats (Takeuchi et al., 2021a). In the following paragraphs, we discuss intervention strategy of epilepsy via MS stimulation for each brain state, based on experimental facts.

During the interictal state (usually resting period), theta rhythm stimulation of the MS can be suggested to reduce seizure susceptibility (Figure 2i). The original idea that theta rhythm activities in the septo-hippocampal axis suppress or oppose epileptic seizures came from the fact that seizure occurrence is less during arousal and REM states (Ng and Pavlova, 2013), when theta band activities dominate. Animal experiments demonstrated that theta rhythm stimulation of the MS increased seizure threshold (decreased seizure susceptibility) in rat and mouse models of TLE (Izadi et al., 2019; Wang et al., 2021). Studies with optogenetic technology suggested that cholinergic tone in the HPC originated from the MS, which decreases during ictal periods, was crucial for the anti-seizure effects of the MS stimulation (Wang et al., 2020b; Takeuchi et al., 2021a). The SST-positive/oriens-lacunosum-moleculare GABAergic interneurons in the HPC presumably mediate the anti-seizure effects by the MS cholinergic signaling (Haam et al., 2018; Wang et al., 2020b). Importantly, the MS-mediated theta rhythm induction in the septo-hippocampal axis can be induced by vagus nerve stimulation (VNS), which is less invasive than DBS (Broncel et al., 2018).

It is noteworthy to mention that the MS stimulation might be employed to prevent development of epileptogenesis after for example traumatic brain injury (Pitk nen et al., 2015). This is because the activation of MS cholinergic neurons during HPC electrical kindling of mice prevented development of seizure susceptibility (Wang et al., 2020b).

**FIGURE 3:**
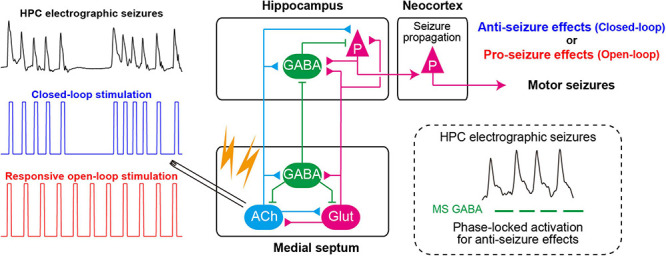
Closed-loop seizure rhythm-driven medial septum (MS) electrical stimulation effectively terminates seizures of HPC origin and suppresses secondary generalization. The precisely timed activation of MS GABAergic neurons may underlie the seizure-terminating effect. In contrast, responsive open-loop MS stimulation does not mitigate (but rather promotes) seizures of HPC origin. ACh, cholinergic neurons; Glut, glutamatergic neurons; HPC, hippocampus (after Takeuchi et al., 2021a).

During the preictal state, the effective strategy would be to decrease seizure susceptibility by inducing theta rhythms in the septo-hippocampal axis by MS rhythm stimulation (or VNS) (Figure 2ii). The preictal state is defined as the time shortly before the onset of an ictal episode when oscillatory brain activities vary from the interictal state. Detecting the preceding changes in oscillatory activities of the brain enables us to predict an upcoming ictal episode and then to prevent development of seizures by intervention (Kuhlmann et al., 2018). The preceding changes in oscillatory activities before ictal episodes can be, for example, global transient increase of entropy in hippocampal and cortical LFPs in rats (Figure 2A) and 1–3 Hz oscillations in the deep posteromedial cortex in humans (Vesuna et al., 2020). The detection of the preceding activities in real time with a closed-loop intervention (brain stimulation) system have already been implemented in the form of the responsive neurostimulation system (RNS^®^ System) in patients, although its stimulation target is not the MS (Morrell, 2011). However, the current detection algorithm of the RNS^®^ System is not perfect and it involves hundreds of false positive detections per day. The unnecessary stimulation of the MS may induce maladaptation in the limbic system and increase seizure susceptibility (kindling effects) (Racine, 1972).

Once seizures have already developed (during ictal states) (Figure 2iii), the responsive MS electrical (or optogenetic) stimulation at a fixed frequency (open-loop) cannot effectively stop seizures (but see Miller et al., 1994; Hristova et al., 2021). Rather, electrical stimulation of the MS at a fixed frequency worsens symptoms of TLE seizures; it induces secondary generalization of partial seizures (Figure 3). We have recently found that closed-loop seizure rhythm stimulation of the MS effectively terminates seizures once they have developed (Takeuchi et al., 2021a; Figure 3). In the study, the LFP in the HPC were continuously monitored with depth electrodes and each MS stimulation was triggered by each deflection of the HPC LFP. The precise stimulus timing of the MS was essential for the seizure-terminating effects; the better that MS stimulation followed the seizure rhythm, the better the seizure-terminating effects were obtained (Takeuchi et al., 2021a).

When ictal episodes have finished, convulsions cease and the LFP traces become flat (postictal state/postictal suppression) (Figure 2iv). Normally, LFPs or EEGs of animals or patients recover within 10 min, and they regain consciousness. However, in severe cases seizure episodes recur and animals or patients cannot recover from the repeated convulsions (status epilepticus). In such emergency cases, the current recommended therapy is administration (preferably intravenous infusion) of benzodiazepine drugs (e.g., diazepam) followed by phenytoin infusion for example (Glauser et al., 2016). Therapeutic effects of the MS stimulation during status epilepticus have not yet been studied.

The DBS electrode in the MS could be used both for theta rhythm stimulation during the preictal (or interictal) state and for seizure rhythm stimulation during the ictal state (Takeuchi et al., 2021a). The same DBS electrode in the MS can serve as a recording electrode because it needs to be implemented as a closed-loop system.

The MS stimulation has been shown to be effective in rodent models of TLE with and without obvious damages of HPC (chronic intrahippocampal kainite model, HPC electrical kindling model), which correspond to human epilepsy with and without sclerosis (Wang et al., 2020b, 2021; Hristova et al., 2021; Takeuchi et al., 2021a). In addition, the MS stimulation has been shown to improve cognitive alterations, which are often comorbid in epilepsy, in animal models of TLE (Izadi et al., 2019; Wang et al., 2021).

The closed-loop on-demand brain stimulation technology has several advantages compared with conventional open-loop DBS: it can be more effective (Morrell, 2011; Berényi et al., 2012; Takeuchi et al., 2021a); it can decrease aversive effects because it does not interfere with normal physiological functions (e.g., learning and memory) or induce maladaptation of the neuronal circuit (e.g., kindling effects) (McIntyre and Gilby, 2009); it prevents development of tolerance; and the therapeutic effects last longer (Shih et al., 2013; Kozák and Berényi, 2017).

### Alzheimer’s Disease

Alzheimer’s disease (AD) is a chronic neurodegenerative disease with well-defined neurological characteristics: amyloid beta plaques, neurofibrillary tangles, and neuronal loss (Takeuchi and Berényi, 2020). AD accounts for nearly 70% of dementia cases worldwide. AD diagnosis is carried out using standardized mental status examinations and the Diagnostic and Statistical Manual of Mental Disorders (DSM-5) (American Psychiatric Association, 2013). EEG for oscillatory disturbances in the brain has emerged as an alternative examination of AD patients (Cassani et al., 2018).

Oscillatory disturbances in the brain have been characterized in AD patients (e.g., decrease of high-frequency components, including gamma-band oscillations). Disruptions of theta oscillations, gamma oscillations and theta–gamma cross-frequency phase–amplitude coupling are commonly observed in the HPC (Goutagny et al., 2013; Ahnaou et al., 2017; Michels et al., 2017; Bazzigaluppi et al., 2018; Wang et al., 2020a) and the EC of various rodent models of AD (Nakazono et al., 2017). The oscillatory disturbances can be causes of cognitive disturbances of AD patients because these oscillations are essential for memory encoding and retrieval. The theta and gamma disruptions in AD are partially due to dysfunctions of SST- and PV-positive interneuron circuits in the HPC, respectively (Chung et al., 2020). In addition, these oscillatory disturbances can originate from disfunctions of the MS in AD because HPC gamma oscillations are modulated by HPC oscillations and HPC oscillations are generated and modulated by the MS cholinergic tone (Butler et al., 2016). There is accumulating evidence for the septal involvement in AD. For example, the number of cholinergic neurons of the basal forebrain, including the MS, was severely decreased in post-mortem brains of AD patients with decreased cholinergic innervation to the HPC (Nelson et al., 2014; Hampel et al., 2018). Amyloid beta injection into the MS induced degeneration of MS cholinergic neurons, disrupted rhythmic activities of MS GABAergic neurons, decreased power of theta oscillations in the HPC, and induced memory deficit of rats (Colom et al., 2010; Villette et al., 2010).

Thus, it is possible to raise a hypothesis that cognitive disfunctions of AD are alleviated by restoring theta and gamma oscillations in the septo-hippocampal axis using DBS.

To date, many preclinical studies have provided evidence that supports this hypothesis. For example, electrical stimulation of the MS (MS-DBS) improved the performance of MS cholinergic neuron-lesioned rats in the Morris water maze task (Jeong et al., 2014, 2017). Chronic electrical stimulation of the fornix (the axonal connection between the MS and the HPC) decreased amyloid beta deposition in the brain of an AD rat model (Leplus et al., 2019). The memory enhancement via the MS-DBS was associated with increased cholinergic signaling in the HPC. Pharmacological enhancement of cholinergic tone by an acetylcholinesterase (AChE) inhibitor restored decreased theta and gamma oscillations and their cross-frequency couplings in the HPC of an AD mouse model (Kumari et al., 2020). The restoration of impaired HPC oscillatory patterns correlated with the improvement of HPC-dependent long-term spatial memory. The relationship between the restoration of healthy oscillatory patterns in the HPC and the memory enhancement might be causal. This is suggested because optogenetic gamma stimulation of PV-positive neurons in the MS during memory retrieval rescued impaired spatial memory in an AD mouse model (J20-APP) (Etter et al., 2019). The MS theta-rhythm stimulation also improved novel object recognition and spatial learning in chronic epileptic models and a traumatic brain injury model in rodents (Lee et al., 2015, 2017; Wang et al., 2021). The theta oscillations in the septo-hippocampal axis can be induce by VNS as well as less invasive stimulation (Broncel et al., 2018).

**FIGURE 4:**
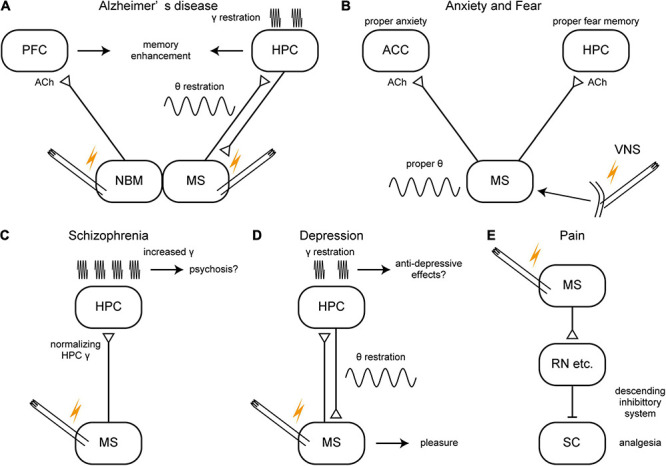
A schema of hypothetical therapeutic strategies of brain disorders via the medial septum. **(A–E)** Potential therapeutic strategies of Alzheimer’s disease, anxiety/fear, schizophrenia, depression, and pain. ACC, anterior cingulate cortex; ACh, acetylcholine; HPC, hippocampus; MS, medial septum; NBM, nucleus basalis of Meynert; PFC, prefrontal cortex; RN, raphe nucleus, SC, spinal cord; VNS, vagus nerve stimulation.

For human applications, the nucleus basalis of Meynert (NBM) and the fornix have already been investigated as DBS target in AD patients with promising outcomes, technical feasibility, and good tolerance (Mirzadeh et al., 2016). DBS of both independently increased glucose metabolism in the brain and improved cognition of patients (Laxton et al., 2010; Kuhn et al., 2015). The stimulation of the NBM may increase cholinergic tone in the brain like that of the MS although their primary target structures are the neocortex and the HPC, respectively (Figure 4A). On the other hand, AChE inhibitors have reached limited success in treating AD patients and the cholinergic neurons degenerate in the NBM of AD patients. Thus, it is not clear whether NMB stimulation would restore healthy oscillations. The stimulation of the fornix would have activated the MS as the fornix is not only a major fiber bundle within the memory circuit of Papez but also the axonal connection between the MS and the HPC. Therefore, together with evidence of animal studies, the MS could be a DBS target for improving or slowing cognitive deficit of AD patients (Figure 4A). Closed-loop phase-specific DBS technology may provide further sophisticated DBS therapies for AD patients (Senova et al., 2018).

Although it is only speculative whether the MS is involved, gamma frequency sensory stimulation has been shown to effectively prevent AD pathology and to improve cognitive functions in animal models of AD (Adaikkan and Tsai, 2020). This finding has been followed by preliminary but promising results of clinical trials (Chan et al., 2021).

### Anxiety/Fear

Chronic and exaggerated anxiety and fear are symptoms of some psychiatric disorders, including generalized anxiety disorder and post-traumatic stress disorders (American Psychiatric Association, 2013). It has been suggested that oscillations in the septo-hippocampal axis are involved in anxiety/fear expression and that the expression is regulated by other limbic networks (Çalışkan and Stork, 2019). For example, increased theta and gamma oscillations within the ventral HPC have been suggested as a biomarker for heightened and impaired fear extinction both in animals and humans. In particular, theta oscillations in the septo-hippocampal axis are suggested to be crucial for anxiety-related behaviors because most anxiolytic (but not anti-psychotic) drugs reduce the frequency of theta oscillations elicited by reticular stimulation; and the immobility-related type 2 theta occurs both during innate predator-elicited arousal/anxiety and during learned anticipatory fear following standard-footshock conditioning (Korotkova et al., 2018).

As noted in section “Roles of the MS in Physiological Oscillations,” many animal studies have provided evidence that the MS is related to anxiety/fear generation and its regulation. For example, electrolytic lesion of the MS, which decreased AChE activity in the HPC, reduced anxiety of rats during successive alleys tests (innate anxiety) and reduced freezing in contextual conditioned fear (learned fear) (Bannerman et al., 2004). Pharmacological inhibition of the MS (with tetrodotoxin, muscimol, or lidocaine infusion) decreased unconditioned and conditioned anxiety (Degroot et al., 2001; Degroot and Treit, 2004; Lamprea et al., 2010), whereas pharmacological activation of the MS (with bicuculline) increased innate anxiety in rats (Ashabi et al., 2011). Cholinergic neurons are involved in MS-mediated anxiety/fear. This is suggested because immunotoxin-mediated ablation or chemogenetic inhibition of the MS cholinergic neurons reduce innate anxiety in mice (e.g., increased time spent in open arms of an elevated plus-maze test) (Nag et al., 2009; Zhang et al., 2017). In contrast, chemogenetic activation of the MS cholinergic neurons reduced theta frequency in the EC and increased innate anxiety in mice (Carpenter et al., 2017). The MS cholinergic neurons are also essential for acquisition, expression, and extinction of fear memory (Knox, 2016). More specifically, MS cholinergic neurons that project to the rostral anterior cingulate cortex, but not those to the ventral HPC, maintain innate (pain-induced) anxiety in mice (Jiang et al., 2018b). On the other hand, MS cholinergic neurons that project to the ventral HPC are required for expression of learned fear in rats (Staib et al., 2018). A knockout and knockdown study suggested that phospholipase C β 4 in the MS is required for maintaining proper levels of cholinergic theta oscillations in the HPC and innate anxiety in mice (Shin et al., 2009). Studies with physostigmine (an AChE inhibitor) also suggested that proper levels of cholinergic tone in the MS or HPC are essential for maintaining proper levels of innate anxiety in rats (Degroot et al., 2001; Sienkiewicz-Jarosz et al., 2003). These reports suggest a possibility that stimulation of MS activity can modify anxiety/fear levels.

DBS has been investigated to alleviate various neurological and psychiatric disorders, including anxiety disorders (Blomstedt et al., 2013; Freire et al., 2020). However, to date, no precisely controlled clinical study with septal stimulation has been conducted to reduce anxiety. Heath and Mickle noted that MS stimulation of patients with intractable pain made patients feel alertness along with an immediate relief of pain and an improved sense of well-being (Gol, 1967; Fisher, 2015). Gol has reported that MS stimulation (4–12 V, 2–5 kHz) in chronic intractable pain patients made patients feel comfortable and relaxed (Gol, 1967). The comfortable state remained up to 24 h after cessation of MS stimulation. A pilot study showed that VNS, which restores normal oscillatory patterns in the septo-hippocampal axis, was effective in alleviating anxiety of treatment-resistant anxiety disorder patients (George et al., 2008; Broncel et al., 2018; Figure 4B).

### Schizophrenia

Schizophrenia is a severe psychiatric disorder characterized by positive symptoms (e.g., delusions, hallucinations, paranoia) and negative symptoms (e.g., loss of motivation, apathy, asocial behavior or loss of affect, poor use and understanding of speech) (American Psychiatric Association, 2013). Schizophrenia patients also have impaired sensorimotor gating and cognitive dysfunctions including disrupted working memory. The symptoms of schizophrenia may stem from typical physiological endophenotypes: the enhanced gamma oscillations and the hyperactive mesolimbic dopamine (DA) system, which are related to the glutamate and the DA hypotheses of schizophrenia, respectively. MS stimulation might normalize the enhanced gamma oscillations in the HPC in humans as well (Figure 4C). The normalized HPC activity might then lead to normalization of the hyperactive mesolimbic DA system. This idea is based on the finding that MS stimulation normalized enhanced gamma oscillations in the HPC of rats and alleviated schizophrenia-like symptoms (Ma and Leung, 2014), and also because the glutamate and DA hypotheses may be bridged with the hyperactive HPC and VTA pathways in schizophrenia (Kätzel et al., 2020).

The glutamate hypothesis is supported by the evidence that systemic administration of NMDA receptor blockers (e.g., phencyclidine, PCP; ketamine) induces schizophrenia-like psychosis in humans (Krystal et al., 1994). The administration of the NMDA receptor blockers induced abnormal gamma oscillations along with psychosis in humans and the abnormal gamma oscillation is one of the endophenotypes of schizophrenia patients (Baldeweg et al., 1998; Lee et al., 2003; Uhlhaas and Singer, 2010). The abnormal gamma oscillations with psychosis by NMDA receptor blockers are presumably elicited by preferential inhibition of NMDA receptors on the PV-positive GABAergic interneurons, which mimics hypofunction of PV-positive GABAergic interneurons in schizophrenia patients (Gonzalez-Burgos and Lewis, 2012). Abnormal gamma oscillations may be suggested as a cause of symptoms of schizophrenia because intervention in the abnormal oscillations with, for example, repeated transcranial magnetic stimulation concomitantly alleviated symptoms of schizophrenia patients (cognitive dysfunctions) (Farzan et al., 2012). The mesolimbic DA hypothesis of schizophrenia originated from clinical observations that symptoms of patients with seizure locus in the midbrain were similar to those of schizophrenia, the fact that amphetamine (a DA transporter blocker) induces schizophrenia-like symptoms, and the fact that blockers of DA D_2_ receptors (neuroleptics) alleviate symptoms of schizophrenia patients, especially positive symptoms (Davis et al., 1991; McCutcheon et al., 2019).

There is accumulating evidence to suggest that the MS is involved in the schizophrenia-like phenotypes in animals. For example, sensorimotor gating deficit is evaluated as prepulse inhibition (PPI) and auditory sensory gating in rodent models, which are closely related to theta and gamma band oscillations in the septo-hippocampal axis (Hajós et al., 2008; Jin et al., 2019). Psychoactive drugs (PCP, ketamine, MK801 or amphetamine) enhanced gamma oscillations in the HPC and induced schizophrenia-like phenotypes in rats (sensory gating deficits, hyperlocomotion). Inactivation of the MS by muscimol-infusion normalized the enhanced gamma oscillations in HPC and alleviated the schizophrenia-like phenotypes induced by the psychoactive drugs (Ma and Stan Leung, 2000; Ma et al., 2004, 2009a, 2012). The enhanced gamma oscillations and altered PPI and auditory gating created by psychoactive drugs in rats were mediated by GABAergic neurons in the MS because they were abolished by ablation of the MS GABAergic neurons by orexin-saporin (Ma et al., 2012). Importantly, DBS of the MS (100 Hz burst stimulation at 16.7% duty cycle) normalized the enhanced gamma oscillations and alleviated the schizophrenia-like phenotypes in ketamine-treated rats (Ma and Leung, 2014).

In the DA hypothesis of schizophrenia, the positive symptoms of schizophrenia are thought to be caused by hyperactivity of midbrain dopaminergic neurons, which is positively modulated by pyramidal neurons in the ventral HPC via NAc and the ventral pallidum (VP) (Sonnenschein et al., 2020). Brain imaging studies of schizophrenia patients have suggested hyperactivity of the anterior HPC, which corresponds to the ventral HPC of rodents (Kätzel et al., 2020; Sonnenschein et al., 2020). The hyperactivity of the ventral HPC (also characterized by enhanced gamma oscillations) leads to hyperactivity of the DA neurons in the VTA via the trisynaptic ventral HPC –> NAc –> VP –> VTA pathway (Sonnenschein et al., 2020). The MS modulates the activity of the ventral HPC. The pharmacological activation of the MS by a local infusion of NMDA induced activation of DA neurons in the VTA via ventral HPC activation in healthy rats (Bortz and Grace, 2018a, b). In contrast, the same activation of the MS leads to inhibition of DA neurons in the VTA in the prenatal methylazoxymethanol (MAM) rats, a rodent model of schizophrenia; this opposite effect is presumably due to hypofunctions of PV-positive interneurons in the ventral HPC in the model (Bortz and Grace, 2018a, b; Sonnenschein et al., 2020). The activation of the MS also alleviated a schizophrenia-like behavioral phenotype in MAM rats (Bortz and Grace, 2018a). Together, these reports suggest that the stimulation of the MS might be beneficial in regulating positive symptoms of schizophrenia by normalizing hyperactive HPC represented with increased gamma oscillations based on the glutamate hypothesis (Figure 4C). In turn, normalizing HPC activity by MS stimulation might normalize HPC –> NAc – > VP –> VTA pathway based on DA hypothesis. However, there is no strong evidence yet to support this idea.

For clinical application, Heath, a psychiatrist of Tulane University, performed initial studies of brain stimulation as a therapy of schizophrenia in the 1950s (Fisher, 2015). His study was based on the hypothesis that schizophrenia is disorder of emotion and stimulation of areas of the brain related to emotion could modulate symptoms of schizophrenia. The MS is one of the areas of the brain believed to be linked to emotions. He found that the patients felt pleasure with the MS stimulation, but the therapeutic outcomes were not favorable (Baumeister, 2000; Fisher, 2015). Fisher pointed out that “The Tulane group had little experience with electrode implantation, and as noted above, initial complication rates were high.” (e.g., infections, seizures) (Fisher, 2015). The MS stimulation might be revisited with current sophisticated DBS technique if the scientific rationale is established for it with an acceptable risk–benefit ratio.

### Depression

Major depressive disorder (MDD) is a common and persistent mental illness with extreme sadness and low mood disproportionate to any possible causes (American Psychiatric Association, 2013). MDD lowers the quality of life of patients and causes a tremendous social burden (Greenberg et al., 2015).

Recent studies have suggested there are oscillatory disturbances in the limbic brain areas of MDD patients and rodent models of depression (Fitzgerald and Watson, 2018; Takeuchi and Berényi, 2020). The oscillatory disturbances are known to be related to symptoms of MDD because the mood reported by patients could be decoded using oscillations in the multiple limbic regions (Reardon, 2017; Sani et al., 2018). They can be utilized as predictors of responses to treatment with antidepressants as well (Baskaran et al., 2012). Furthermore, the symptoms of MDD have been alleviated by interventions of the abnormal oscillations in patients (Noda et al., 2017; Reardon, 2017).

Recent advances of biological studies have shown that the oscillations in the septo-hippocampal axis are affected by depression and involved in its symptoms. For example, olfactory bulbectomy, a model of depression, decreased the number of cholinergic neurons in the MS (Kang et al., 2010). Systemic administration of an antidepressant drug (reboxetine, a norepinephrine reuptake inhibitor) increased theta power and gamma power in the HPC and increased theta phase-locking of septal-unit activities (Hajós et al., 2003). MS is also known as the pleasure center of the brain (Olds and Milner, 1954; Bishop et al., 1963). Studies investigating MS’s relationship to rewarding and pleasure raise a possibility that stimulation of the MS might be effective for alleviating symptoms of MDD (Figure 4D). Although pharmacological treatments become dominant after the discovery of the first antidepressant, imipramine (Kuhn, 1958), DBS f has been revisited for patient with MDD resistant to pharmacological treatments (Mayberg et al., 2005). DBS of the medial forebrain bundle has been already employed for the patients of treatment-resistant depression and revealed to be effective (Dandekar et al., 2018). The anti-depressive effects of the medial forebrain bundle stimulation may be mediated by the activation of the MS because the rewarding effects of the medial forebrain bundle encourage rats to repeated self-stimulation (Olds and Milner, 1954) and septal lesions attenuate this effect (Jacques, 1979; Fisher, 2015).

The feeling caused by MS electrical stimulation has been reported in earlier studies of depression, epilepsy, schizophrenia, and refractory pain patients (Bishop et al., 1963; Gol, 1967; Schvarcz, 1993). Their reports included “good,” “well-being,” “relaxed,” or “pleasurable” feelings, which can be built up to a sexual orgasm (Heath, 1972; Moan and Heath, 1972). They successfully alleviated depressed states of patients by septal stimulation. However, the euphoria induced by septal stimulation can be addictive in both humans and animals and can cause repeated self-administration (stimulation) until they become exhausted (Olds and Milner, 1954; Bishop et al., 1963). Therefore, it is important to limit availability of stimulation to avoid addiction by setting appropriate stimulus parameters (e.g., maximum number of stimulations, minimum duration of interstimulus interval) (Oshima and Katayama, 2010).

### Pain

Pain is an important function that alerts individuals to, for example, a tissue injury with nociception and unpleasant feelings. Pain normally disappears when the tissue injury is cured. However, if pain persists and becomes chronic, the chronic pain (e.g., neuropathic pain) significantly decreases the quality of life of patients. The tremendous pain of, for example, cancer patients with continuous tissue invasion should be properly controlled as well. Existing therapy, including analgesic drugs (such as narcotics, non-steroidal anti-inflammatory drugs, analgesic adjuvant), cannot control every type of pain, including chronic and continuous pain. Therefore, DBS has been investigated for those treatment-resistant types of pain (Levy, 2003; Bittar et al., 2005; Pereira et al., 2014). The septum has been one of the targets for DBS for intractable pain.

The MS is a part of the pain system in the brain. The MS receives afferents from the nociceptive system/pathway (e.g., the spinal cord) and an electrophysiological study showed that more than 50% neurons in the MS are activated by peripheral nociceptive stimulation (Dutar et al., 1985; Burstein et al., 1987). Another study showed that chronic peripheral inflammation induced by complete Freund’s adjuvant induces c-Fos expression in the MS neurons. Approximately 70% of the c-Fos-positive MS neurons were cholinergic neurons and the remaining were glutamatergic or GABAergic neurons (Jiang et al., 2018a).

Accumulating evidence from rodent studies has implicated the MS in both processing and regulation of pain (Ang et al., 2017). For encoding of pain-related memory, the theta oscillations in the septo-hippocampal axis are essential to acquire the memory of the pain-induced negative affects. The peripheral nociceptive stimulation (e.g., hind paw injection of formalin, noxious heat stimulation on the tail) induced theta oscillations in the septo-hippocampal pathway, and electrical lesion of the MS attenuated the sensory-evoked type 2 theta oscillations in the HPC suggesting that the MS transmits pain-related information to the HPC (Khanna, 1997). The nociception-induced theta oscillations increased signal-to-noise ratio of sensory-evoked firing of pyramidal neurons in the HPC CA1 area for processing of nociceptive information (Zheng and Khanna, 2001). The selective lesion of either MS GABAergic or cholinergic neurons disrupted the nociception-induced theta oscillations in the HPC (Ang et al., 2015). Attenuation of the nociception-induced theta oscillations by deleting GABAergic signaling in the MS disrupted the memory of the pain-induced negative affect. However, the attenuation of the nociception-induced theta oscillations did not significantly decrease formalin-induced nociceptive behaviors of mice (Ang et al., 2015).

The MS has roles in the regulation of pain as well. On one hand, the MS maintains awareness of pain. This idea is supported by the evidence that inhibition of the MS by muscimol (a GABA_A_ receptor agonist) or AMPA/NMDA antagonist reduced experimental neuropathic pain of mice (Ariffin et al., 2018) and that infusion of muscimol or zolpidem (an allosteric modulator of GABA_A_ receptors) suppressed formalin-induced licking and flinching (Lee et al., 2011). Inactivation or lesion of the MS also prolonged analgesic effects of general anesthesia (Ma et al., 2002; Leung et al., 2013).

On the other hand, the MS controls exaggerated pain. Importantly, it is known that electrical stimulation of the MS inhibited the firing rate of wide dynamic range neurons in the spinal cord dorsal horn evoked by the peripheral noxious stimulation (pressure, pinch, heat) in anesthetized rats and cats (Carstens et al., 1982; Hagains et al., 2011). Those analgesic effects induced by the MS electrical stimulation are supposed to be mediated by activation of the descending pain inhibitory system (Figure 4E). Recent studies have revealed that selective inhibition of the MS cholinergic neurons with chemogenetic technology attenuates nociceptive behaviors of mice models of chronic inflammatory pain. The MS cholinergic neurons projecting the rostral anterior cingulate cortex are hyperactive in the chronic inflammatory state, and selective inhibition of the pathway induced the same analgesic effects (Jiang et al., 2018a). On the other hand, the MS cholinergic neurons projecting the ventral HPC are hypoactive, and selective activation of the pathway induced analgesic effects in the pain model (Jiang et al., 2018a). This report suggested that whether activation of MS cholinergic neurons inhibit or facilitate pain is dependent on their projections.

In humans, Heath and Mickle found that septal stimulation induced an immediate relief of chronic pain in patients (Fisher, 2015). In 1967, Gol studied the effect of the MS on his six severe pain patients (Gol, 1967). In the case of one of his patients, the patient had satisfactory analgesia by septal stimulation (4–12 V peak-to-peak, 20–60 μs duration at 2–5 kHz). The patient had severe cancer pain from metastatic lesions in the spine and the hip, but he felt no pain and was comfortable while being stimulated. The analgesic effect was not frequency dependent between 2 and 5 kHz but was stimulus intensity-dependent. In the other cases, septal stimulation partially alleviated their severe pain. They felt comfort with the septal stimulation, although the pain was still perceived. The analgesic effect with the septal stimulation persisted from several hours to 24 h after the stimulation. However, only one patient out of six cases with multiple electrode insertions in the septum had satisfactory relief of pain. The septal stimulation was well-tolerated by all six patients. Schvarcz also reported the analgesic effect of septal electrical stimulation. Twelve of 19 implanted patients experienced partial relief of pain by septal stimulation (Schvarcz, 1993). It was noted that low-intensity septal stimulation induced pain relief and higher-intensity stimulation induced a feeling of well-being and relaxation. Tasker pointed out that septal stimulation gave rise to feelings of flushing, paresthesia, nausea, nystagmus and a feeling of warmth (Tasker, 1982). Stimulation of the MS can exert an inhibitory effect on access to the spinothalamic tract (Tasker, 1982) as suggested by animal experiments (Carstens et al., 1982; Hagains et al., 2011).

## Author Contributions

YT and AB developed the idea. YT, AN, LB, and QL prepared the figures. YT, AN, and LB wrote the original draft. MO, KM, and AB discussed and commented on the manuscript. All authors contributed to the article and approved the submitted version.

## Conflict of Interest

AB and AJN are the owner of Amplipex Llc. and a shareholder of Neunos Ltd., Szeged, Hungary, manufacturers of signal-multiplexed neuronal amplifiers and neurostimulator devices. The remaining authors declare that the research was conducted in the absence of any commercial or financial relationships that could be construed as a potential conflict of interest.

## Abbreviations

ACh: acetylcholine
AChE: acetylocholinesterase
AD: Alzheimer’s disease
AMPA: α-amino -3-hydroxy-5-methyl-4-isoxazolepropionic acid
CA1: field CA1 of the cornu ammonis
CA3: field CA3 of the cornu ammonis
CaBP: calbindin
ChAT: choline acetyltransferase
CR: calretinin
DA: dopamine
DBS: deep brain stimulation
DG: dentate gyrus
DSM-5, Diagnostic and Statistical Manual of Mental Disorders: Fifth Edition
EC: entorhinal cortex
EEG: electroencephalography
GAD: glutamic acid decarboxylase
HCN: hyperpolarization-activated cyclic nucleotide-gated
HDB: horizontal limb of diagonal band
HPC: hippocampus
LEC: lateral entorhinal cortex
LFP: local field potential
LS: lateral septum
MAM: methylazoxymethanol
MDD: major depressive disorder
MEC: medial entorhinal cortex
MRN: median raphe nucleus
MS: medial septum
NAc: nucleus accumbens
NBM: nucleus basalis of Meynert
NI: nucleus incertus
NMDA: *N*-methyl -D-aspartate
PCP: phencyclidine
PPI: prepulse inhibition
PV: parvalbumin
REM: rapid eye movement
SST: somatostatin
SPW–R: sharp wave-ripple
SuM: supramammillary nucleus
TLE: temporal lobe epilepsy
TMN: tuberomammillary nucleus
VDB: vertical limb of diagonal band
VGluT: vesicular glutamate transporter
VNS: vagus nerve stimulation
VP: ventral pallidum
VTA: ventral tegmental area.
